# 547 ADHD and Burn Injuries: The Hidden Readmission Risk in Pediatric Patients

**DOI:** 10.1093/jbcr/irae036.181

**Published:** 2024-04-17

**Authors:** Alexandra Halevi, Melanie Anaya, Brent Emigh, David Harrington, Tareq Kheirbek

**Affiliations:** Alpert Medical School at Brown University, Providence, RI; Brown University Alpert Medical School, Providence, RI; Alpert Medical School at Brown University, Providence, RI; Brown University Alpert Medical School, Providence, RI; Alpert Medical School at Brown University, Providence, RI; Brown University Alpert Medical School, Providence, RI; Alpert Medical School at Brown University, Providence, RI; Brown University Alpert Medical School, Providence, RI; Alpert Medical School at Brown University, Providence, RI; Brown University Alpert Medical School, Providence, RI

## Abstract

**Introduction:**

Attention-deficit/hyperactivity disorder (ADHD) is a common neurodevelopmental disorder with the hallmark characteristics of poor impulse control, inattention, hyperactivity, and other cognitive dysfunction; as a consequence, children with ADHD are at an increased risk of burn injury. We hypothesized that pediatric burn patients with ADHD have a higher rate of unplanned readmission compared to patients without a diagnosis of ADHD.

**Methods:**

We analyzed the Nationwide Readmission Database (01/2018 - 10/2018) to compare 60-days readmission rates in children (< 18 yo) with and without a diagnosis of ADHD who were treated for burn injuries on their index admission. Weighted multiple logistic regression was performed to obtain population estimates of odds ratios (OR), adjusting for patients’ demographics, burn severity and location, comorbidities, socioeconomic status, other psychiatric illnesses, and burn treatment on index admission.

**Results:**

Our cohort included 1,879 children with burn injury. Of these, 79 (4.2%) had a diagnosis of ADHD. ADHD patients were more likely to be male (86.1% vs 59.8%, p=0.0002 ) and older (11.6 ± 3.7 vs 5.4 ± 5.3 years, p=0.0001). Children with ADHD were more likely to have other psychiatric diagnoses (26.6% vs 3.8%), specifically obsessive compulsive disorder (2.5% vs 0%, p=0.004), and other developmental disorders (15.2% vs 1.6%, p=0.0002). Patients with ADHD were more likely to be readmitted within 60 days of their index admission (15.2% vs. 4.8%, p=0.002). On weighted multiple regression analysis, ADHD had a higher adjusted odds ratio of readmission (OR: 2.66, 95%CI: 1.13-6.29).

**Conclusions:**

Children with ADHD have a higher adjusted risk of readmission following thermal injury. This is potentially due to higher barriers to compliance with wound care, physical therapy, and scar management. Targeted discharge planning for patients with ADHD, including scheduled short-term follow-up appointments, home nursing assistance, and post-discharge phone calls, may potentially lower readmission risks in this vulnerable population.

**Applicability of Research to Practice:**

Pediatric patients with ADHD are at higher than average risk for unplanned readmission within 60 days of discharge. Pediatric burn centers have the opportunity to adjust their post-discharge practice with these high-risk patients to decrease this risk. Interventions could include: more frequent clinic follow-up, longer duration with outpatient therapy, and additional coaching for caretakers on how to improve patient compliance.

